# Internal structure validity of the Brazilian version of the Health Professional Education in Patient Safety Survey

**DOI:** 10.1590/0034-7167-2024-0323

**Published:** 2026-08-03

**Authors:** Ana Carolina Simões Pereira, Maria do Carmo Fernandez Lourenço Haddad, Sonia Silva Marcon, Elena Bohomol, Flávio Rebustini, Laura Misue Matsuda

**Affiliations:** I Universidade Estadual de Maringá. Maringá, Paraná Brazil; II Universidade Estadual de Londrina. Londrina, Paraná, Brazil; III Universidade Federal de São Paulo. São Paulo, São Paulo, Brazil; IV Universidade de São Paulo. São Paulo, São Paulo, Brazil

**Keywords:** Patient Safety, Teaching, Nursing, Validation Study, Surveys and Questionnaires., Seguridad del Paciente, Enseñanza, Enfermería, Estudio de Validación, Encuestas y Cuestionarios.

## Abstract

**Objectives:**

to assess the internal structure validity of the Brazilian version of the Health Professional Education in Patient Safety Survey among nursing students.

**Methods:**

a psychometric study was conducted with 402 final-year nursing students from 17 universities. Measurement properties were tested using Exploratory Factor Analysis and Item Response Theory, combined with cross-validation. Reliability was assessed using Cronbach’s Alpha and McDonald’s Omega indicators. Data analysis was performed using IBM^®^ SPSS Statistics 20.0 and Factor^®^ software.

**Results:**

the unidimensional 16-item model explained 60.76% of the variance in clinical practice and 55.25% in classroom settings. Factor loadings, communalities, and item discrimination were adequate, with stability and replicability maintained in cross-validation.

**Conclusions:**

the Brazilian version of the instrument demonstrated evidence of internal structure validity among nursing students, contributing to the identification of gaps in patient safety education.

## RESUMEN

**Objetivos:**

evaluar la validez de la estructura interna de la versión brasileña del instrumento *Health Professional Education in Patient Safety Survey* entre estudiantes de Enfermería.

**Métodos:**

estudio psicométrico realizado con 402 estudiantes del último año del curso de Enfermería de 17 universidades. Las propiedades de medición fueron evaluadas mediante Análisis Factorial Exploratorio y Teoría de Respuesta al Ítem, asociadas a la validación cruzada. La confiabilidad se evaluó utilizando los indicadores Alfa de Cronbach y Omega de McDonald. Para el análisis de los datos se utilizaron los programas *IBM^®^ SPSS Statistics 20.0 y Factor^®^*.

**Resultados:**

el modelo unidimensional de 16 ítems explicó el 60,76% de la varianza en la práctica clínica-asistencial y el 55,25% en el contexto de aula. Las cargas factoriales, comunalidades y discriminación de los ítems fueron adecuadas, con estabilidad y capacidad de replicación mantenidas en la validación cruzada.

**Conclusiones:**

la versión brasileña del instrumento presentó evidencias de validez de estructura interna entre estudiantes de Enfermería, lo que contribuye a identificar las brechas en el aprendizaje sobre seguridad del paciente.

## INTRODUCTION

Internationally, the healthcare landscape is negatively impacted by incidents associated with the care provided to the population. As such, addressing the topic of patient safety in the training of human resources has emerged as a key strategy to ensure the safety and quality of care, beginning with technical and/or academic education and continuing throughout professional development^(1,2)^. In Brazil and around the world, barriers to its implementation are still observed, such as limited coverage in curricula, lack of engagement and interest from faculty, and weaknesses in the management and planning of education^(3,4)^.

Regarding patient safety, with the aim of enhancing the competencies of future professionals and providing theoretical support for curriculum development, the World Health Organization published in 2011 the Patient Safety Curriculum Guide: Multi-professional Edition. Its structure is also based on the theoretical framework of the Canadian Patient Safety Institute, which outlines six sociocultural competencies for patient safety: 1 - Working in teams with other healthcare professionals; 2 - Effective communication; 3 - Safety risk management; 4 - Understanding human and environmental factors; 5 - Recognition, response, and disclosure of adverse events and near misses and 6 - Safety culture^(1,4)^.

The Canadian framework mentioned served as the theoretical basis for the Health Professional Education in Patient Safety Survey (H-PEPSS), an instrument developed to assess the self-reported confidence of students and newly graduated healthcare professionals regarding sociocultural competencies in patient safety, both in clinical practice and classroom settings, given that learning in the health field occurs through academic engagement in both environments^(4,5)^.

Regarding the use of measurement instruments, the H-PEPSS has been tested and validated in countries and cultural contexts such as Saudi Arabia^(6,7)^, Iran^(8)^, India^(9)^, Belgium^(10)^, Cyprus/Greece^(11)^, Turkey^(12)^, China^(13)^, Italy^(14)^, and South Korea^(15)^. It has also been used in studies conducted in other countries including Canada^(16-18)^, South Korea^(19)^, Italy^(20,21)^, Australia^(22)^, the United States^(23,24)^, Saudi Arabia^(25,26)^, and Iran^(27)^, with validity evidence tested in Saudi Arabia, Iran, India, Belgium, Cyprus/Greece, Turkey, China, Italy, and South Korea^(6-15)^.

For use in different countries, research instruments must undergo adaptation and validation according to the cultural specificities of each nation. To achieve this, the application of contemporary psychometric techniques is recommended, supported by the Standards for Educational and Psychological Testing from institutions specialized in instrument validity, an increasingly technical and scientific requirement in the healthcare field^(28)^.

Given the need for studies and broad dissemination of patient safety competencies in multiprofessional education, it is important to enable the assessment and evaluation of this topic using reliable and validated research instruments^(29)^.

## OBJECTIVES

To assess the internal structure validity of the Brazilian version of the H-PEPSSbr instrument among nursing students.

## METHODS

### Ethical aspects

The ethical principles of Resolution 466/2012 were respected. This research project was approved by the Research Ethics Committee involving Human Subjects at the *Universidade Estadual de Maringá* (State University of Maringá). Informed consent was obtained from all individuals participating in the study, either online or in person. For the cross-cultural adaptation process and validation evidence assessment, authorization was granted by Prof. Dr. Liane R. Ginsburg, the lead author of the original study^(5)^.

### Study design, period, and setting

This is a psychometric study developed from the stage of verifying the measurement properties of the H-PEPSSbr, based on the Standards for Educational and Psychological Testing. It was conducted between August 2021 and October 2022, using a purposive sample of 402 nursing students. All participants were enrolled in the final year of their undergraduate nursing program at 17 Higher Education Institutions (HEIs) across five Brazilian states: São Paulo, Paraná, Minas Gerais, Mato Grosso do Sul, and Rio Grande do Sul.

### Data collection

This investigation was conducted in two formats: online and in person. In the online format, data collection was carried out using Google Forms^®^, made publicly accessible through the dissemination of an invitation and an informational video via the study’s website and Instagram^®^, as well as through WhatsApp^®^ groups and contacts that included nursing faculty and students. To facilitate data collection, an invitation letter was also sent to the coordinators of nursing programs, based on a search for email addresses on university websites.

### Data collection instruments

The instrument titled “Survey on Health Professional Education in Patient Safety - H-PEPSSbr Brazilian version” was used, along with a brief questionnaire developed by the researcher containing variables related to sociodemographic data and familiarity with patient safety education.

The H-PEPSS was initially adapted into Brazilian Portuguese through a postgraduate (Master’s level) study conducted by the researcher^(30)^, following the guidelines for the cross-cultural adaptation of self-report measures^(28)^, which include: I) Translation; II) Synthesis of translations; III) Back translation; IV) Review by an expert committee; V) Pre-testing, and VI) Evaluation of the cross-cultural adaptation process. In stage IV of the aforementioned study, an additional step was included to assess evidence of content validity and response process.

The H-PEPSSbr includes an instruction section for respondents, states the objectives of the instrument, and presents the conceptual definition of patient safety. It consists of 38 items divided into three sections, evaluated using a five-point Likert scale ranging from “strongly disagree” to “strongly agree,” with an additional option “I don’t know what this is about.” Only the first section, which addresses the sociocultural dimensions of patient safety, was validated^(5)^.

The test’s dimensionality is structured as follows: D1: Clinical/assistive safety (4 items); D2: Teamwork with other healthcare professionals (3 items); D3: Effective communication (3 items); D4: Safety risk management (3 items); D5: Understanding human and environmental factors (2 items); D6: Recognition, response, and disclosure of adverse events and near misses (2 items); D7: Safety culture (3 items). Among these, the first dimension is presented exclusively to help respondents focus on clinical competencies and subsequently distinguish and direct their attention to the six sociocultural competencies (D2-D7)^(5)^.

### Participants

Regarding the assessment of the internal structure of instruments, samples larger than 400 are recommended, along with a minimum ratio of ten cases per variable-item, ideally 20:1^(32-33)^. Considering a validated instrument composed of 16 items extracted from a 23-item version, related to six sociocultural dimensions of patient safety, the minimum sample size was established at 230 respondents. However, a target of at least 400 participants was chosen to ensure robust evidence of the instrument’s internal structure, as recommended by contemporary psychometric literature^(32)^. Participants were undergraduate nursing students, regularly enrolled in the final year of their program during the 2020-2021 academic period.

### Data analysis and processing

To assess evidence of internal structure validity, a combination of Exploratory Factor Analysis (EFA) and Item Response Theory (IRT) techniques was employed, along with cross-validation procedures^(34,35)^.

To evaluate factorability, sample adequacy indices were applied to the full set of 23 items for EFA, including the Kaiser-Meyer-Olkin (KMO) test, Bartlett’s test of sphericity, and the matrix determinant. For the KMO test, a minimum value between 0.80 and 0.90 was considered acceptable for factorability^(36)^. Bartlett’s test of sphericity was expected to yield a p-value < 0.05^(33)^, and the matrix determinant was expected to exceed p > 0.00001^(36)^.

Dimensionality analysis was conducted using Robust Parallel Analysis (RPA), specifically the Optimal Implementation of Parallel Analysis (PA) with Minimum Rank Factor Analysis, which minimizes the common residual variance^(37)^. Test robustness was determined through bootstrap^(38)^ procedures and sample extrapolation to 5,000 cases.

In both learning contexts, the reduced version of the H-PEPSSbr was also tested using two additional techniques to strengthen dimensionality evidence: the Bayesian Information Criterion (BIC)^(39)^ and a newer factor retention method known as the Hull Method (HULL)^(40)^. Subsequent testing proceeded with the reduced 16-item version and its corresponding cross-validation.

To enhance the quality of dimensionality evidence, indicators were adopted to examine whether the set of items demonstrates unidimensional characteristics: Unidimensional Congruence (UNICO) > 0.95, Explained Common Variance (ECV) > 0.80, and Mean of Item Residual Absolute Loadings (MIREAL) < 0.30^(41)^.

Regarding factor extraction, the Robust Unweighted Least Squares (RULS) method was used, which allows for matrix rotation and is more suitable for data that do not follow a normal distribution^(42,43)^. The Oblimin rotation method was employed, based on the assumption that factors would be correlated^(33)^.

For item retention or exclusion, minimum acceptable factor loadings were set between 0.30 and 0.40^(33)^. Acceptable communality, considering sample size and high factor loadings^(44)^, was defined as a minimum of 0.30. For model-explained variance, a minimum threshold of 50% was assumed.

To strengthen the evidence of internal structure, Item Response Theory was applied using the Normal-Ogive Graded Response Model (NOGRM), which measures the strength of association between each item and the latent variable to assess the adequacy of factor loadings. Item discrimination parameter “a” was evaluated, with acceptable values defined as follows: 0.65-1.35 (moderate discrimination), 1.35-1.69 (high discrimination), and >1.70 (very high discrimination)^(45-47)^.

Additionally, regarding the EFA, the G-H index was used to assess model replicability, with acceptable values above 0.80^(48)^. For factor quality and estimation, marginal reliability based on the Expected A Posteriori (EAP) method was applied, with values greater than 0.80 considered acceptable for the reliability of instrument scores^(41)^.

Finally, to enhance the reliability and replicability of the proposed model, cross-validation was performed^(29)^. The datasets were designated as follows: H-PEPSSbr in the classroom: Sample 1 (full dataset A - 402 cases), Sample 2 (training dataset A - 201 cases), Sample 3 (test dataset A - 201 cases). H-PEPSSbr in clinical practice: Sample 1 (full dataset B - 402 cases), Sample 2 (training dataset B - 201 cases), Sample 3 (test dataset B - 201 cases)

For reliability analysis, Cronbach’s Alpha and McDonald’s Omega were used, with values equal to or greater than 0.70 considered acceptable^(50)^. All analyses were conducted using IBM^®^ SPSS Statistics 20 and Factor^®^ version 12.02.01.

## RESULTS

A total of 128 respondents participated in the online data collection and 298 in the in-person collection, totaling 426, of which 402 final-year Nursing students were deemed eligible. Students from 17 HEIs were included. There was a predominance of students enrolled in HEIs located in the state of Paraná, 349 (86.8%), with most, 346 (86.0%), enrolled in four-year programs. The average age was 24.9 years, with a predominance of female students, 334 (83.1%), single individuals, 314 (78.1%), and family income ranging from 2 to 3 minimum wages, 156 (38.3%), and from 4 to 5 minimum wages, 104 (25.8%).

Regarding variables related to exposure to Patient Safety Education, the majority, 346 (86.7%), reported having had a specific class on the topic during their training, and only 154 (50.6%) stated that they had a dedicated course on patient safety. As for projects involving the subject, most reported not having participated in any (302 = 75.7%). A total of 340 (85.2%) students stated that patient safety should be a subject within the course curriculum.

Preliminary tests were conducted to explore the H-PEPSSbr data in both its full (23 items) and reduced (16 items) versions. It was observed that both versions showed sample adequacy. The 23-item H-PEPSSbr dataset for the classroom scenario presented a Kaiser-Meyer-Olkin (KMO) index of 0.84, Bartlett’s sphericity = 4,092.8 (df = 253; p < 0.0001), and a matrix determinant < 0.000001. Therefore, the data were analyzed using the validated 16-item version.

Regarding the dimensionality testing of the 16-item dataset, based on the theoretical framework of the six-dimension model, in addition to confirming one-dimensionality, issues/non-conformities related to cross loadings were identified. Exceptions included items 1, 7, 10, and 11 for the classroom setting, and items 7, 10, and 12 for the clinical-practice setting. Problems related to item discrimination were also observed, with most items scoring below 0.65 in both scenarios. Further details of this process are available in the supplementary technical report. The explained variance for the latent variable (sociocultural competencies in patient safety) in the classroom scenario for the full dataset A was 55.25%, in the training dataset it was 56.67%, and in the test dataset, 52.05%. In the clinical-practice setting, the full dataset B achieved 60.76%, the training dataset 59.90%, and the test dataset 56.56%.

Extensive dimensionality testing was confirmed through the application of UNICO, ECV, and MIREAL values, with indices supporting one-dimensionality in the full dataset and in the cross-validation subsamples, as shown in Table 1.

**Table 1:** Values of Unidimensional Congruence (UNICO), Explained Common Variance (ECV), and Mean of Item Residual Absolute Loadings (MIREAL) from the Brazilian version of the Health Professional Education in Patient Safety Survey (H-PEPSSbr), for classroom and clinical practice settings, with testing applied to the full and cross-validation samples, Maringá, Paraná, Brazil, 2025

Testing	H-PEPSSbr^*^ (classroom) 16 itens			H-PEPSSbr^*^ (clinical practice) 16 itens		
**Sample**	**UNICO** ^†^	**ECV** ^‡^	**MIREAL** ^§^	**UNICO** ^†^	**ECV** ^‡^	**MIREAL** ^§^
Full Dataset	0.96	0.86	0.23	0.98	0.88	0.24
Training Dataset	0.96	0.86	0.26	0.98	0.89	0.23
Dataset	0.98	0.88	0.23	0.98	0.89	0.21

^^*^^H-PEPSSbr - Versão brasileira do Health Professional Education in Patient Safety Survey; ^†^UNICO - Unidimensional Congruence;

‡ ECV - Explained Common Variance;

§ MIREAL - Mean of item residual absolute loadings.

The fact that the instrument is unidimensional eliminated the need for rotational techniques in the factor matrix, indicating the application of the Normal-Ogive graded response model from Item Response Theory (IRT), which is appropriate for a unidimensional polytomous model.

Table 2 presents the values of factor loadings (λ), communalities (h^2^), and item discrimination (a) for the full sample and two cross-validation subsamples of the H-PEPSSbr in the classroom setting.

**Table 2:** Values of factor loadings, communalities, and item discrimination from the unidimensional Brazilian version of the Health Professional Education in Patient Safety Survey (H-PEPSSbr) in the classroom setting, Maringá, Paraná, Brazil, 2025

Item	H-PEPSSbr^*^ (classroom)								
	Training Dataset A			Test Dataset A			Full Dataset		
	λ†	h2^‡^	a§	λ†	h2^‡^	a§	λ†	h2^‡^	a§
1	0.56	0.32	0.68	0.59	0.35	0.73	0.57	0.33	0.70
2	0.66	0.44	0.89	0.62	0.38	0.79	0.66	0.43	0.88
3	0.65	0.43	0.87	0.59	0.35	0.74	0.63	0.40	0.81
4	0.80	0.65	1.36	0.73	0.54	1.09	0.79	0.62	1.28
5	0.78	0.60	1.24	0.82	0.67	1.43	0.81	0.65	1.38
6	0.67	0.45	0.92	0.62	0.39	0.80	0.64	0.41	0.83
7	0.76	0.58	1.19	0.72	0.52	1.04	0.73	0.54	1.08
8	0.79	0.62	1.30	0.82	0.68	1.45	0.80	0.64	1.34
9	0.75	0.57	1.16	0.83	0.69	1.51	0.78	0.62	1.27
10	0.58	0.34	0.72	0.61	0.38	0.78	0.60	0.36	0.75
11	0.64	0.41	0.83	0.72	0.52	1.06	0.68	0.46	0.93
12	0.66	0.44	0.88	0.54	0.29	0.65	0.60	0.36	0.75
13	0.76	0.57	1.17	0.72	0.52	1.06	0.74	0.55	1.11
14	0.77	0.60	1.23	0.71	0.51	1.02	0.74	0.55	1.12
15	0.80	0.63	1.33	0.69	0.48	0.96	0.76	0.58	1.18
16	0.81	0.66	1.39	0.66	0.44	0.88t	0.75	0.57	1.16

^^*^^H-PEPSSbr - Brazilian version of the Health Professional Education in Patient Safety Survey; †λ - Factor loadings; ‡h^2^ - Communalities; §a - Discrimination.

Factor loadings for the full dataset A of the H-PEPSSbr ranged from 0.57 to 0.81; in the training dataset A, from 0.56 to 0.81; and in the test dataset A, from 0.54 to 0.83. This analysis did not detect any issues related to collinearity/multicollinearity or Heywood cases, and the loading values were considered acceptable.

Regarding communalities, values in the full dataset A ranged from 0.33 to 0.65; in the training dataset A, from 0.32 to 0.66; and in the test dataset A, from 0.29 to 0.69. It is worth noting that for item 12, the communality value was 0.299, which is at the expected threshold. As for item discrimination, values ranged from 0.70 to 1.38 in the full dataset A, from 0.68 to 1.39 in the training dataset A, and from 0.65 to 1.51 in the test dataset A, with discrimination classified as moderate to high.

Table 3 presents the values of factor loadings (λ), communalities (h^2^), and item discrimination (a) for the full sample and two cross-validation subsamples of the H-PEPSSbr in the clinical practice setting.

**Table 3:** Values of factor loadings, communalities, and item discrimination from the unidimensional Brazilian version of the Health Professional Education in Patient Safety Survey (H-PEPSSbr) in the clinical practice, Maringá, Paraná, Brazil, 2025

Item	H-PEPSSbr^*^ (clinical practice)								
	Training Dataset B			Teste Dataset B			Full Dataset B		
	λ†	h2^‡^	a§	λ†	h2^‡^	a§	λ†	h2^‡^	a§
1	0.60	0.36	0.76	0.69	0.48	0.96	0.64	0.42	0.85
2	0.81	0.65	1.38	0.72	0.52	1.04	0.76	0.58	1.19
3	0.73	0.53	1.07	0.71	0.51	1.03	0.72	0.52	1.05
4	0.81	0.66	1.40	0.76	0.59	1.19	0.78	0.61	1.27
5	0.79	0.63	1.30	0.80	0.65	1.37	0.79	0.62	1.28
6	0.76	0.59	1.20	0.72	0.53	1.06	0.74	0.56	1.13
7	0.71	0.51	1.03	0.70	0.49	0.98	0.70	0.49	0.99
8	0.82	0.67	1.45	0.74	0.55	1.11	0.78	0.61	1.26
9	0.79	0.63	1.32	0.72	0.52	1.04	0.76	0.58	1.19
10	0.66	0.43	0.88	0.66	0.44	0.89	0.69	0.48	0.96
11	0.77	0.60	1.12	0.74	0.54	1.10	0.76	0.58	1.17
12	0.58	0.33	0.71	0.66	0.44	0.90	066	0.43	0.88
13	0.64	0.41	0.83	0.84	0.70	1.15	0.73	0.54	1.09
14	0.75	0.56	1.13	0.70	0.49	0.99	0.72	0.52	1.05
15	0.75	0.57	1.16	0.77	0.59	1.21	0.76	0.58	1.17
16	0.71	0.51	1.02	0.69	0.48	0.97	0.70	0.49	0.98

* >H-PEPSSbr - Brazilian version of the Health Professional Education in Patient Safety Survey;

† λ - Factor loadings;

‡ h^2^ - Communalities;

§ a - Discrimination.

In the full dataset B of the H-PEPSSbr, factor loadings ranged from 0.64 to 0.79; in the training dataset B, from 0.58 to 0.82; and in the test dataset B, from 0.66 to 0.84. At this stage, no issues related to collinearity/multicollinearity or Heywood cases were detected, and the loading values were considered acceptable.

Regarding communalities, values in the full dataset B ranged from 0.42 to 0.62; in the training dataset B, from 0.36 to 0.67; and in the test dataset B, from 0.44 to 0.70 - all within the expected range.

As for item discrimination, values ranged from 0.85 to 1.28 in the full dataset B, from 0.71 to 1.45 in the training dataset B, and from 0.89 to 1.37 in the test dataset B, with discrimination classified as moderate to high.

Reliability indicators for the H-PEPSSbr, both for full dataset A and full dataset B, showed Cronbach’s alpha and McDonald’s omega values of 0.94. In the cross-validation, training dataset A presented both Cronbach’s alpha and McDonald’s omega of 0.94, while in the test dataset A, both indicators remained at 0.93. In the cross-validation for training and test datasets B, both indices remained at 0.94. These reliability indices demonstrated satisfactory model fit, as did the factor loadings, communalities, and item discrimination values for both the full samples and the cross-validation subsamples.

Regarding construct replicability based on the latent and observed G-H index, the values found for full dataset A ranged from 0.94 to 0.80; for training dataset A, from 0.91 to 0.82; and for test dataset A, from 0.94 to 0.79, respectively. It is worth noting that the observed G-H index in test dataset A was approximately 0.80 (0.797), which is at the threshold. For full dataset B, the indices ranged from 0.95 to 0.84; for training dataset B, from 0.95 to 0.83; and for test dataset B, from 0.95 to 0.84, respectively.

Regarding the quality and effectiveness of the model’s score estimation, the Expected A Posteriori (EAP) reliability index was 0.95 in the classroom setting and 0.94 in the clinical practice setting, for the full datasets A and B. In training dataset A, it was 0.95, and in test dataset A, 0.94. For both training and test datasets B, the EAP value was 0.95, all considered adequate.

**Figure 1:**
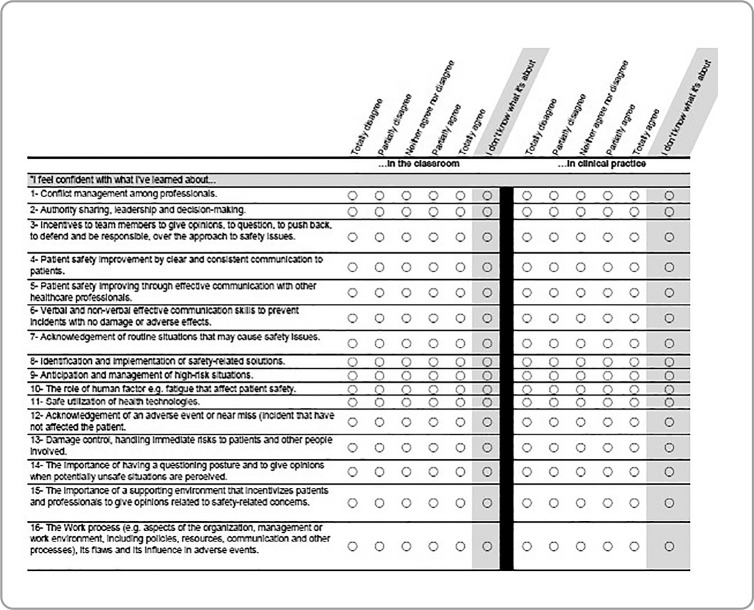
Unidimensional structure of the items from the Brazilian version of the Health Professional Education in Patient Safety Survey (H-PEPSSbr*), Maringá, Paraná, Brazil, 2020.

It is evident that the Brazilian version of the H-PEPSSbr provides internal structural evidence supporting a unidimensional model capable of assessing nursing students’ self-reported confidence in learning sociocultural competencies related to patient safety, as illustrated in Figure 1.

## DISCUSSION

The initial verification of the Exploratory Factor Analysis (EFA), which includes the KMO index, Bartlett’s test of sphericity, and the determinant of the matrix, showed satisfactory indices for the H-PEPSSbr. In the literature, there are assumptions that the use of these indicators supports the continuation of factor analysis, provided the values meet acceptable thresholds^(33)^.

In integrating EFA with Item Response Theory, the comparative model for dimensionality, between the reduced version (16 items) and the full version (23 items), revealed a unidimensional structure through Parallel Analysis. In the original study, the authors justified the use of Confirmatory Factor Analysis (CFA) instead of EFA with the argument that “a confirmatory factor analysis approach was considered more appropriate than exploratory factor analysis”^(5)^. However, this claim lacks technical support in advanced psychometric literature, which actually offers extensive recommendations emphasizing the importance of model exploration through EFA^(5)^.

In testing both the reduced and full versions of the six-dimension model, as proposed by the theoretical framework, issues were identified such as the presence of cross-loadings and unacceptable item discrimination, in both the classroom and clinical practice settings. According to the literature, cross-loadings are problematic and provide evidence of model inadequacy^(34)^, as they indicate that a given item loads acceptably onto more than one dimension/factor, suggesting overlap between distinct constructs. In this context, such items should be considered for retention or extraction from the matrix^(33)^.

Item discrimination also yielded values below acceptable thresholds. These results suggest that the six-dimension model does not adequately measure the strength of association between each item and the latent variable. One-dimensionality was confirmed through Parallel Analysis, supported by the combination of BIC and HULL methods. Notably, the dimensionality testing of the reduced version of the H-PEPSSbr, when combined with these methods, reinforced the unidimensional structure of the model.

The literature indicates that PA is particularly suitable for data that do not follow a normal distribution^(42,43)^, which is common in Likert-type scales. However, combining PA with other techniques enhances the robustness of dimensionality analyses, as demonstrated in this study. BIC enables model approximation based on Bayesian statistics, allowing comparisons between hypothetical models^(39)^, while HULL seeks a balance between model fit and degrees of freedom^(40)^.

This investigation employed contemporary and comprehensive techniques, including indicators that are not commonly used in psychometric studies. Some of these indicators, such as UNICO, ECV, and MIREAL, are relatively recent and not available in commercial software. The use of cross-validation also enabled a robust analysis of the initial data exploration, based on an independent dataset that strongly guided the analytical process^(51)^.

Within the scope of the model’s factor loadings, the reduced unidimensional version of the H-PEPSSbr did not present issues or inconsistencies related to multicollinearity or Heywood cases. Multicollinearity occurs when factor loadings exceed 0.85, which may indicate item redundancy, data distribution problems, and distortions in the measurement of the latent variable. Heywood cases^(28)^, on the other hand, arise when loadings fall outside the -1 to +1 range, typically associated with sample inadequacies, improper estimation of error variance, and model indeterminacy. The absence of these interpretative biases allows us to affirm that no harmful effects related to sample or model were observed.

Item discrimination, based on the application of Item Response Theory, showed satisfactory indices, confirming the reliability of the instrument and reinforcing the validity of the results obtained through factor analysis indicators. From a reliability perspective, Cronbach’s alpha and McDonald’s omega values were satisfactory, ranging from 0.93 to 0.94. The Cronbach’s alpha values found in this study were higher than those reported in Ginsburg’s study^(5)^, which ranged from 0.81 to 0.85 across the six reported dimensions.

In this study, Exploratory Factor Analysis, or the unrestricted model, aimed to identify a structure within a set of items without establishing a priori relationships regarding the number of components to be extracted^(32)^. The use of IRT integrated with other techniques enabled the combination of multiple evaluation methods through robust procedures^(35)^. This approach allows for assessing the likelihood of model stability across studies, populations, or subpopulations^(41)^.

Marginal reliability via Expected A Posteriori also indicated values that reflect the reliability of the instrument’s scores. According to the literature, satisfactory EAP indices demonstrate the quality of the score estimates produced by the instrument^(41,49)^. However, it is important to note that further testing is needed regarding the test’s parameterization

In one study^(11)^, a final instrument consisting of 38 items was presented, while in three other studies^(8,12,14)^, the H-PEPSS was presented with 23 items, indicating various configurations of the instrument and the use of different validity indices, some of which show methodological weaknesses. There was exclusive use of Cronbach’s alpha^(11)^; CFA combined with external, convergent, and discriminant validation^(9)^; CFA with discriminant validation^(10)^; CFA alone^(5,12,15)^; EFA^(14)^; and a combination of CFA and EFA^(8,13)^.

### Study limitations

The Brazilian version of the H-PEPSSbr is the first study to report a unidimensional structure, supported by contemporary techniques that demonstrate the internal structural validity of this model. The limitations related to sociocultural competencies, still not widely integrated into our cultural context, may be associated with the nonconformity of a multidimensional model in the Brazilian version. The lack of other scales designed for the same purpose within the Brazilian context currently represents a limitation of this study for potential comparisons. It is recommended that, following the consolidation of sociocultural competencies in patient safety within the Brazilian reality, further efforts be made to explore the validity of associations with other variables and the consequences of the test.

### Contribution to the field of Nursing

The internal structure testing of the Brazilian version of the H-PEPSSbr demonstrated that the instrument is capable of assessing nursing students’ self-reported confidence regarding sociocultural competencies in patient safety. It provided evidence supporting a unidimensional model with satisfactory reliability and item discrimination. This instrument serves as an important tool for planning and/or monitoring actions aimed at promoting patient safety within higher education institutions and continuing education settings. Moreover, it highlights the institutional need to include this topic in the multiprofessional health curriculum within the national cultural context, in a more consistent and standardized manner.

## CONCLUSIONS

The Brazilian version of the H-PEPSSbr demonstrated a unidimensional structure, showing validity evidence for internal consistency among nursing students, with indications of replicability across other educational contexts in the health field. The integration of contemporary techniques reflects scientific advancement and originality in the validation process.

## Data Availability

The research data are not available.
