# Supplementary material for: Internal structure validity of the Brazilian version of the Health Professional Education in Patient Safety Survey
Source: Rev Bras Enferm. 2026 Aug 3;79:e20240323. doi: 10.1590/0034-7167-2024-0323 (PMC13435864; doi:10.1590/0034-7167-2024-0323)
Supplement: Translation

# Translation: Validade da estrutura interna da versão brasileira do *Health Professional Education in Patient Safety Survey*

**DOI:** 10.1590/0034-7167-2024-0323pt

**Authors:** Ana Carolina Simões Pereira, Maria do Carmo Fernandez Lourenço Haddad, Sonia Silva Marcon, Elena Bohomol, Flávio Rebustini, Laura Misue Matsuda

**Affiliations:** I Universidade Estadual de Maringá. Maringá, Paraná Brasil; II Universidade Estadual de Londrina. Londrina, Paraná, Brasil; III Universidade Federal de São Paulo. São Paulo, São Paulo, Brasil; IV Universidade de São Paulo. São Paulo, São Paulo, Brasil

**Keywords:** Segurança do Paciente, Ensino, Enfermagem, Estudos de Validação, Inquéritos e Questionários.

## Abstract

**Objetivos:**

avaliar a validade da estrutura interna da versão brasileira do instrumento *Health Professional Education in Patient Safety Survey* entre estudantes de Enfermagem.

**Métodos:**

estudo psicométrico realizado com 402 estudantes do último ano do curso de Enfermagem de 17 universidades. As propriedades de medida foram testadas com Análise Fatorial Exploratória e Teoria de Resposta ao Item, associadas à validação cruzada. A avaliação da confiabilidade usou os indicadores *Alfa de Cronbach* e *Ômega de McDonald*. Para a análise dos dados, utilizaram-se os softwares IBM^®^ *SPSS Statistics* 20.0 e *Factor* ^®^.

**Resultados:**

o modelo unidimensional de 16 itens obteve variância explicada de 60,76% na prática clínico-assistencial e 55,25% em sala de aula. As cargas fatoriais, comunalidades e discriminação dos itens foram adequadas, com estabilidade e replicabilidade mantidas na validação cruzada.

**Conclusões:**

a versão brasileira do instrumento apresentou evidências de validade da estrutura interna entre estudantes de Enfermagem, de modo a contribuir na identificação das lacunas do aprendizado de segurança do paciente.

## INTRODUÇÃO

Internacionalmente, o cenário da assistência à saúde é impactado negativamente devido a incidentes associados aos cuidados ofertados à população. Dessa forma, a abordagem sobre o tema de segurança do paciente na formação de recursos humanos se caracteriza como estratégia em voga à garantia da segurança e da qualificação dos cuidados, desde a formação técnica e/ou acadêmica e, também, em âmbito contínuo^(1,2)^. No Brasil e no mundo, ainda são observadas barreiras à sua efetivação, como pouca abordagem nos currículos, falta de adesão e interesse dos professores, e fragilidade na gestão e planejamento do ensino^(3,4)^.

No que tange à segurança do paciente, no sentido de melhorar as competências dos futuros profissionais como subsídio teórico à construção de currículos, em 2011 foi publicado o guia da Organização Mundial da Saúde (OMS), intitulado, “*Patient safety curriculum guide: multiprofessional edition”*, cuja estrutura se pauta também no marco teórico do *Canadian Patient Safety Institute* (CPSI), que aborda seis competências socioculturais de segurança do paciente, a saber: 1- Trabalho em equipe com outros profissionais de saúde; 2- Comunicação efetiva; 3-Gerenciamento de riscos de segurança; 4-Compreensão de fatores humanos e ambientais; 5-Reconhecimento, resposta e revelação (*disclosure*) de eventos adversos e *near miss* (incidente que não atingiu o paciente); e 6- Cultura de segurança^(1,4)^.

O marco canadense referido foi o arcabouço teórico do *Health Professional Education in Patient Safety Survey* (H-PEPSS), instrumento criado para avaliar a confiança autorrelatada de estudantes e profissionais de saúde recém-formados no contexto das competências socioculturais de segurança do paciente, na prática clínico-assistencial e em sala de aula, visto que o aprendizado na área de saúde decorre da atuação acadêmica em ambos os cenários^(4,5)^.

No que se refere ao processo de uso de instrumentos de medida, o H-PEPSS foi testado e validado em países e realidades culturais como Arábia Saudita^(6,7)^, Irã^(8)^, Índia^(9)^ ,Bélgica^(10)^, Chipre/Grécia^(11)^, Turquia^(12)^, China^(13)^, Itália^(14)^ e Coreia do Sul^(15)^. O H-PEPSS foi utilizado também em estudos realizados em outros países: Canadá^(16-18)^, Coréia do Sul^(19)^, Itália^(20,21)^, Austrália^(22)^, Estados Unidos^(23,24)^, Arábia Saudita^(25,26)^ e, ainda, no Irã^(27)^, com as evidências de validade testadas em países como a Arábia Saudita, Irã, Índia, Bélgica, Chipre/Grécia, Turquia, China, Itália e Coréia do Sul^(6-15)^.

Para uso em diferentes países, instrumentos de pesquisas necessitam ser submetidos à adaptação e validação nas especificidades culturais de cada nacionalidade. Para isto, é recomendado o emprego de técnicas da psicometria contemporânea, com aporte de *Standards for Educational and Psychological Testing* de instituições especializadas em validade de instrumentos, o que tem se tornado uma exigência técnico-científica na área de saúde^(28)^.

Diante da necessidade de estudos e ampla divulgação sobre as competências de segurança do paciente na educação multiprofissional, faz-se importante oportunizar a verificação/avaliação do tema, com uso de instrumentos de pesquisa confiáveis e validados^(29)^.

## OBJETIVOS

Avaliar a validade da estrutura interna da versão brasileira do instrumento H-PEPSSbr entre estudantes de Enfermagem.

## MÉTODOS

### Aspectos éticos

Foram respeitados os preceitos éticos da Resolução 466/2012. O projeto desta pesquisa foi aprovado pelo Comitê de Ética em Pesquisa envolvendo Seres Humanos da Universidade Estadual de Maringá. O Consentimento Livre e Esclarecido foi obtido de todos os indivíduos envolvidos no estudo por meio *online* ou presencial. Para a realização do processo *cross-cultural* e verificação de evidências de validade, foi concedida autorização pela Profa. Dra. Liane R. Ginsburg, autora principal do estudo original^(5)^.

### Desenho, período e local de estudo

Trata-se de um psicométrico, desenvolvido a partir da etapa de verificação das propriedades de medida do H-PEPSSbr, baseado no *Standards for Educational and Psychological Testing* ^(28)^. realizado entre os meses de agosto de 2021 a outubro de 2022, com uma amostra intencional de 402 estudantes em Enfermagem. Estavam matriculados no último ano do curso em 17 Instituições de Ensino Superior (IES) de cinco estados Brasileiros: São Paulo, Paraná, Minas Gerais, Mato Grosso do Sul e Rio Grande do Sul.

### Coleta de dados

Esta investigação foi desenvolvida de duas formas, *online* e presencial. No formato *online*, a pesquisa foi realizada por meio do *Google Forms^®^,* em livre acesso público, pela divulgação de convite e vídeo informativo pelo *site* e *Instagram^®^* do estudo, além de grupos e contatos de *WhatsApp^®^* que continham docentes e discentes de Enfermagem. Para operacionalizar a coleta, também foi encaminhada uma carta-convite aos coordenadores dos cursos de Enfermagem, por meio de uma busca de endereços eletrônicos realizada nos Sites de Universidades.

### Instrumentos de coleta de dados

Foi empregado o instrumento intitulado “Pesquisa sobre Educação de Profissionais da Saúde em Segurança do Paciente H-PEPSSbr versão brasileira”, e um breve instrumento contendo variáveis relacionadas a dados sociodemográficos e de aproximação com o Ensino de Segurança do Paciente, criado pelo pesquisador.

O H-PEPSS foi inicialmente adaptado para o português Brasileiro, por intermédio de um estudo realizado em nível de Pós-Graduação (Mestrado) pela pesquisadora^(30)^, que seguiu as diretrizes propostas para o processo de adaptação transcultural de medidas de autorrelato^(31)^, quais sejam: I) Tradução; II) Síntese das traduções; III) *Back translation*; IV) Revisão por um comitê de especialistas; V) Pré-teste e VI) Avaliação do processo de adaptação transcultural. No estágio IV) da referida pesquisa, foi incluída uma etapa de avaliação das evidências de validade de conteúdo e de processo resposta.

O H-PEPSSbr conta com tópico de instruções aos respondentes, enuncia os objetivos do instrumento e apresenta a definição conceitual de segurança do paciente. É composto por 38 itens e três seções, avaliadas em uma escala do tipo *Likert* de cinco pontos, que varia de “discordo fortemente” a “concordo fortemente”, incluindo uma opção “não sei do que se trata”, e sendo validada apenas a primeira seção referente às dimensões socioculturais de segurança do paciente^(5)^.

A dimensionalidade do teste é apresentada da seguinte maneira: D1- Segurança clínica/assistencial (4 itens); D2 - Trabalho em equipe com outros profissionais de saúde (3 itens); D3 - Comunicação efetiva (3 itens); D4 - Gerenciamento de riscos de segurança (3 itens); D5 -Compreensão de fatores humanos e ambientais (2 itens); D6 - Reconhecimento, resposta e revelação (*disclosure*) de eventos adversos e *near miss* (incidente que não atingiu o paciente) (2 itens); D7 - Cultura de segurança (3 itens). Desses, a primeira dimensão é apresentada exclusivamente para os respondentes manterem o foco de atenção às competências clínicas e, posteriormente, conseguirem distinguir e direcionar o olhar às seis competências socioculturais (D2-D7)^(5)^.

Participantes

No tocante à avaliação da estrutura interna de instrumentos, recomendam-se amostras acima de 400, assim como um padrão mínimo de dez casos para cada variável-item de instrumento, idealmente 20:1^(32,33)^. Contando com um instrumento validado que possui 16 itens extraídos de uma versão de 23 itens, relacionados às seis dimensões socioculturais de segurança do paciente, a amostra mínima foi estabelecida em 230 respondentes. Entretanto, optou-se pelo alcance mínimo de 400 participantes para verificação da evidência da estrutura interna do instrumento, segundo recomendam publicações da psicometria contemporânea^(32)^. Participaram estudantes de Graduação em Enfermagem, regularmente matriculados no último ano do curso, no período letivo de 2020-2021.

### Tratamento e análise de dados

Para avaliação das evidências de validade de estrutura interna, optou-se pela integração de técnicas da Análise Fatorial Exploratória (AFE) e Teoria de Resposta ao Item (TRI), associadas à validação cruzada^(34,35)^.

Com o intuito de avaliar a fatorabilidade foram aplicados os índices de adequação da amostra completa para a AFE (23 itens), por meio do teste de *Kaiser-Meyer-Olkin* (KMO), do teste de esfericidade de Bartlett, além do determinante de matriz. Para o KMO, considerou-se um valor mínimo de 0,80 a 0,90 (bom) para fatorabilidade^(36)^. No teste de esfericidade de Bartlett, foi esperado valor de p < 0,05^(33)^. Em relação à investigação do determinante da matriz, estabeleceram-se valores p > 0,00001^(36)^.

A análise da dimensionalidade do teste por meio da Análise Paralela Robusta (APR), utilizando-se a *Optimal Implementation of Parallel Analysis* (PA) com *Minimum rank factor analysis* minimiza a variância comum dos resíduos^(37)^. A robustez do teste foi determinada com base na associação de um *bootstrap* ^(38)^ e com uma extrapolação amostral para 5.000.

Para ambos os cenários de aprendizagem, a versão reduzida do H-PEPSSbr também foi testada com mais duas técnicas, visando ampliar as evidências de dimensionalidade, quais sejam: a *Bayesian Information Criterion* (BIC)^(39)^ e um novo método de retenção fatorial, denominado *Hull Method* (HULL)^(40)^. Posteriormente, o seguimento das testagens se deu com a versão reduzida (de 16 itens) e com a consequente validação cruzada.

Ainda, para agrupamento de técnicas que conduzem à melhor qualidade das evidências de dimensionalidade, adotaram-se indicadores que examinam se o conjunto de itens apresenta característica unidimensional: *Unidimensional Congruence* (UNICO) >0,95, *Explained Common Variance* (ECV) >0,80, e *Mean of Item Residual Absolute Loadings* (MIREAL) <0,30^(41)^.

No tocante à extração, utilizou-se a *Robust Unweighted Least Square* (RULS), que tem potencial de rotação da matriz e é mais indicada para dados que não seguem a normalidade^(42,43)^. Como método de rotação, foi empregada a *Oblimin*, com a prerrogativa de que os fatores se correlacionariam^(33)^.

Para a retenção ou exclusão de itens, consideraram-se aceitáveis cargas fatoriais mínimas entre 0,30-0,40^(33)^. A comunalidade aceitável, associada ao tamanho da amostra, foi definida para o mínimo de 0,30 diante do tamanho amostral e do carregamento de cargas fatoriais elevadas^(44)^. Já para a variância explicada do modelo, pressupôs-se valor mínimo de 50%.

Com o intuito de qualificar as evidências da estrutura interna, adotou-se a técnica TRI associada à *Normal-Ogive Graded Response Model (NOGRM)*, que mede a força de associação entre o item e a variável latente, para avaliação da adequação das cargas fatoriais. Empregou-se a avaliação do item de discriminação “a”, considerando aceitáveis valores entre 0,65 e 1,35 (discriminação moderada), 1,35-1,69 (alta discriminação), e >1,70 (discriminação muito alta)^(45-47)^. Ainda, referente à AFE, utilizou-se o índice G-H para analisar a replicabilidade do modelo, com valores aceitáveis acima de 0,80^(48)^. Já no tocante à qualidade e estimativa dos fatores, empregouse a confiabilidade marginal da Expectativa *a Posteriori* (EAP), considerando valores maiores que 0,80 como aceitáveis para a confiabilidade dos *scores* dos instrumentos^(41)^.

Por fim, para aumentar a confiabilidade e a replicabilidade do modelo proposto, aplicou-se a validade cruzada^(49)^. Os bancos foram, assim, denominados: H-PEPSSbr na sala de aula, amostra 1 (banco completo A - 402 casos), amostra 2 (banco treinamento A - 201 casos), amostra 3 (banco teste A - 201 casos), e H-PEPSSbr na prática clínico-assistencial, amostra 1 (banco completo B - 402 casos), amostra 2 (banco treinamento B - 201 casos) e amostra 3 (banco teste B - 201 casos).

Dentre os indicadores para análise da confiabilidade, utilizou-se o *Alfa de Cronbach* e *Ômega* de *MCDonald,* cujos valores iguais ou acima de 0,7 foram considerados aceitáveis^(50)^. As análises foram efetuadas por meio do IBM^®^ SPSS *Statistics* 20 e F*actor^®^ 12.02.01*.

## RESULTADOS

Participaram 128 respondentes na coleta *online* e 298 na coleta presencial, totalizando 426, dos quais, foram elegíveis 402 estudantes do último ano do curso em Enfermagem. Contou-se com estudantes de 17 IES. Houve predominância de alunos/estudantes matriculados em IES localizadas no estado do Paraná, 349 (86,8%), dos quais a maioria 346 (86,0%) era de cursos estruturados em quatro anos. A média de idade foi de 24,9 anos, com predominância do sexo feminino, 334 (83,1%), solteiros, 314 (78,1%), com renda familiar entre 2 a 3 salários mínimos, 156 (38,3%) e com 4 a 5 salários mínimos, 104 (25,8%).

No que diz respeito às variáveis de aproximação com o Ensino de Segurança do Paciente, a maioria, 346 (86,7%), afirmou ter tido uma aula específica sobre o tema durante a formação, e apenas 154 (50,6%) referiram possuir uma disciplina específica de segurança do paciente. Em relação a projetos que envolvessem o assunto, a maioria acusou não ter participado de nenhum (302=75,7%). Um total de 340 (85,2%) alunos afirmou que a segurança do paciente deveria ser uma disciplina na unidade curricular do curso.

Foram realizados testes preliminares no intuito de explorar os dados do H-PEPSSbr nas suas versões completa (23 itens) e reduzida (16 itens). Observou-se que ambas as versões apresentaram resultados de adequação da amostra. O banco de 23 itens do H-PEPSSbr, para a sala de aula, apresentou índice de *Kaiser-Meyer-Olkin* (KMO) = 0,84, esfericidade de *Bartlett* = 4.092,8 (df =253; p < 0,0001) e determinante da matriz < 0,000001. Assim, os dados foram analisados seguindo a versão validada de 16 itens.

No tocante à testagem da dimensionalidade do banco com 16 itens, baseado no arcabouço teórico do modelo para seis dimensões, além de se constatar a unidimensionalidade, foram identificados problemas/não conformidades referentes a *cross loadings*. Excetuaram-se apenas os itens 1, 7, 10 e 11 para sala de aula, e os itens 7, 10, e 12 para a prática clínico-assistencial. Constataram-se, também, problemas referentes à discriminação na maioria dos itens que estiveram abaixo de 0,65 para ambos os cenários. O maior detalhamento deste processo se encontra no relatório técnico complementar. A variância explicada para a variável latente (competências socioculturais de segurança do paciente), para o cenário de sala de aula no banco completo A foi de 55,25%, no banco treinamento se situou em 56,67%, e no banco teste, em 52,05%. Na prática clínica assistencial, o banco completo B obteve 60,76%, o banco treinamento, 59,90%. e o banco teste, 56,56%.

Confirmou-se a testagem extensiva da dimensionalidade com a aplicação dos valores de UNICO, para o ECV e o MIREAL, com índices que atestaram para a unidimensionalidade no banco completo e nas subamostras da validação cruzada, conforme constam na Tabela 1.

**Tabela 1:** Valores de *Unidimensional Congruence* (UNICO), *Explained Common Variance* (ECV) e *Mean of Item Residual Absolute Loadings* (MIREAL), da versão brasileira do *Health Professional Education in Patient Safety Survey* (H-PEPSSbr) para os ambientes sala de aula e prática clínicoassistencial com testagem para a amostra completa e cruzada, Maringá, Paraná, Brasil, 2025

Testagem	H-PEPSSbr^*^ (sala de aula) 16 itens			H-PEPSSbr^*^ (prática clínicoassistencial) 16 itens		
**Amostra**	**UNICO** ^†^	**ECV** ^‡^	**MIREAL** ^§^	**UNICO** ^†^	**ECV** ^‡^	**MIREAL** ^§^
Banco completo	0,96	0,86	0,23	0,98	0,88	0,24
Banco Treinamento	0,96	0,86	0,26	0,98	0,89	0,23
Banco	0,98	0,88	0,23	0,98	0,89	0,21

^^*^^H-PEPSSbr - Versão brasileira do Health Professional Education in Patient Safety Survey; †UNICO - Unidimensional Congruence;

‡ ECV - Explained Common Variance;

§ MIREAL - Mean of item residual absolute loadings.

O fato de o instrumento ser unidimensional dispensou a realização de técnicas rotacionais da matriz fatorial, indicando a aplicação da técnica de *Normal-ogive graded response model* da TRI, adequada para o modelo unidimensional politômico.

Na Tabela 2, constam os valores das cargas fatoriais (**λ)**, comunalidades (h2) e discriminação (a) do item para a amostra completa e duas subamostras cruzadas para o H-PEPSSbr na sala de aula.

**Tabela 2:** Valores de cargas fatoriais, comunalidades e discriminação dos itens da versão brasileira unidimensional do *Health Professional Education in Patient Safety Survey* (H-PEPSSbr) na sala de aula, Maringá, Paraná, Brasil, 2025

Itens	H-PEPSSbr^*^ (sala de aula)								
	Banco treinamento A			Banco teste A			Banco completo A		
	λ†	h2^‡^	a§	λ†	h2^‡^	a§	λ†	h2^‡^	a§
1	0,56	0,32	0,68	0,59	0,35	0,73	0,57	0,33	0,70
2	0,66	0,44	0,89	0,62	0,38	0,79	0,66	0,43	0,88
3	0,65	0,43	0,87	0,59	0,35	0,74	0,63	0,40	0,81
4	0,80	0,65	1,36	0,73	0,54	1,09	0,79	0,62	1,28
5	0,78	0,60	1,24	0,82	0,67	1,43	0,81	0,65	1,38
6	0,67	0,45	0,92	0,62	0,39	0,80	0,64	0,41	0,83
7	0,76	0,58	1,19	0,72	0,52	1,04	0,73	0,54	1,08
8	0,79	0.62	1,30	0,82	0,68	1,45	0,80	0,64	1,34
9	0,75	0,57	1,16	0,83	0,69	1,51	0,78	0,62	1,27
10	0,58	0,34	0,72	0,61	0,38	0,78	0,60	0,36	0,75
11	0,64	0,41	0,83	0,72	0,52	1,06	0,68	0,46	0,93
12	0,66	0,44	0,88	0,54	0,29	0,65	0,60	0,36	0,75
13	0,76	0,57	1,17	0,72	0,52	1,06	0,74	0,55	1,11
14	0,77	0,60	1,23	0,71	0,51	1,02	0,74	0,55	1,12
15	0,80	0,63	1,33	0,69	0,48	0,96	0,76	0,58	1,18
16	0,81	0,66	1,39	0,66	0,44	0,88t	0,75	0,57	1,16

^^*^^H-PEPSSbr - Versão brasileira do Health Professional Education in Patient Safety Survey;

† λ - Cargas fatoriais;

‡ h2 - Comunalidades;

§ a - Discriminação.

As cargas fatoriais para o banco completo A do H-PEPSSbr se situaram entre 0,57 a 0,81, no banco treinamento A, entre 0,56 e 0,81, e no banco teste A, entre 0,54 e 0,83. Nesta análise, não foram detectados problemas de colinearidade/multicolinearidade e *heywood cases,* e os valores das cargas se apresentaram aceitáveis.

Em relação às comunalidades, no banco completo A, os valores se situaram entre 0,33 a 0,65, no banco treinamento A, entre 0,32 a 0,66, e no banco teste A, entre 0,29 e 0,69. Ressalta-se que no tocante ao item 12, a comunalidade referida se remete a 0,299, portanto, no limite esperado. No que se refere à discriminação dos itens, os valores estiveram entre 0,70 a 1,38 no banco completo A, entre 0,68 e 1,39 no banco treinamento A, e entre 0,65 e 1,51 no banco teste A, com discriminação classificada entre alta e moderada.

Na Tabela 3, são apresentados os valores das cargas fatoriais (**λ)**, comunalidades (h2) e discriminação (a) do item para a amostra completa e duas subamostras cruzadas para o H-PEPSSbr na prática clínico-assistencial.

**Tabela 3:** Valores de cargas fatoriais, comunalidades e discriminação dos itens da versão brasileira unidimensional do *Health Professional Education in Patient Safety Survey* (H-PEPSSbr) na prática clínico-assistencial, Maringá, Paraná, Brasil, 2025

Itens	H-PEPSSbr^*^ (prática clínico-assistencial)								
	Banco treinamento B			Banco teste B			Banco completo B		
	λ†	h2^‡^	a§	λ†	h2^‡^	a§	λ†	h2^‡^	a§
1	0,60	0,36	0,76	0,69	0,48	0,96	0,64	0,42	0,85
2	0,81	0,65	1,38	0,72	0,52	1,04	0,76	0,58	1,19
3	0,73	0,53	1,07	0,71	0,51	1,03	0,72	0,52	1,05
4	0,81	0,66	1,40	0,76	0,59	1,19	0,78	0,61	1,27
5	0,79	0,63	1,30	0,80	0,65	1,37	0,79	0,62	1,28
6	0,76	0,59	1,20	0,72	0,53	1,06	0,74	0,56	1,13
7	0,71	0,51	1,03	0,70	0,49	0,98	0,70	0,49	0,99
8	0,82	0,67	1,45	0,74	0,55	1,11	0,78	0,61	1,26
9	0,79	0,63	1,32	0,72	0,52	1,04	0,76	0,58	1,19
10	0,66	0,43	0,88	0,66	0,44	0,89	0,69	0,48	0,96
11	0,77	0,60	1,12	0,74	0,54	1,10	0,76	0,58	1,17
12	0,58	0,33	0,71	0,66	0,44	0,90	066	0,43	0,88
13	0,64	0,41	0,83	0,84	0,70	1,15	0,73	0,54	1,09
14	0,75	0,56	1,13	0,70	0,49	0,99	0,72	0,52	1,05
15	0,75	0,57	1,16	0,77	0,59	1,21	0,76	0,58	1,17
16	0,71	0,51	1,02	0,69	0,48	0,97	0,70	0,49	0,98

^^*^^H-PEPSSbr - Versão brasileira do Health Professional Education in Patient Safety Survey;

† λ - Cargas fatoriais;

‡ h2 - Comunalidades;

§ a - Discriminação.

No banco completo B do H-PEPSSbr, as cargas fatoriais se situaram entre 0,64 e 0,79, entre 0,58 e 0,82 no banco treinamento B, e entre 0,66 e 0,84 no banco teste B. Nesta etapa, também não foram detectados problemas de colinearidade/multicolinearidade e *Heywood cases,* considerando-se que os valores das cargas eram aceitáveis.

Em relação às comunalidades, no banco completo B, os valores ficaram entre 0,42 a 0,62, no banco treinamento B entre 0,36 a 0,67, e no banco teste B, entre 0,44 e 0,70, todos no limite esperado. No tocante à discriminação dos itens, este se situou entre 0,85 a 1,28 no banco completo B, entre 0,71 e 1,45 no banco treinamento B, e entre 0,89 e 1,37 no banco teste B, com discriminação classificada entre alta e moderada.

Os valores indicativos de confiabilidade para o H-PEPSSbr tanto para o banco completo A como para o banco completo B, os índices de *Alfa de Cronbach* e *ômega de McDonald,* resultaram em 0,94. Na validação cruzada, o banco treinamento A apresentou *Alfa de Cronbach* e Ômega de *McDonald* de 0,94, e no banco teste A, ambos os indicadores se mantiveram em 0,93. Na validação cruzada, no banco treinamento e teste B, os dois índices se mantiveram em 0,94. Os índices de confiabilidade apresentaram valores satisfatórios de ajuste ao modelo, assim como as cargas fatoriais, comunalidades e discriminação de itens, tanto para as amostras completas como para as subamostras da validação cruzada.

Quanto à replicabilidade do construto pelo índice G-H latente e observado, os índices encontrados para o banco completo A se estabeleceram entre 0,94 a 0,80, no banco treinamento A entre 0,91 a 0,82, e 0,94 a 0,79 no banco teste A, respectivamente. Vale ressaltar que o índice G-H observado no banco teste A se refere ao valor aproximado de 0,80 (0,797), ou seja, limiar. Já para o banco completo B, os índices se situaram entre 0,95 a 0,84, no banco treinamento B de 0,95 a 0,83, e no banco teste B entre 0,95 a 0,84, respectivamente.

No tocante à qualidade e efetividade da solução dos escores do modelo, o índice EAP se situou em 0,95 na sala de aula, e em 0,94 na prática clínico-assistencial, nos bancos completos A e B. No banco treinamento A esteve em 0,95 e teste A 0,94. No banco de treinamento e teste B, o EAP resultou em 0,95, ambos sendo considerados adequados.

Constata-se que a versão brasileira do H-PEPSSbr acumula evidências de estrutura interna para um modelo unidimensional capaz de avaliar a confiança autorrelatada de estudantes de enfermagem a respeito do aprendizado das competências socioculturais de segurança do paciente, conforme constam na Figura 1.

**Figura 1:** Estrutura unidimensional dos itens da versão brasileira do *Health Professional Education in Patient Safety Survey* (H-PEPSSbr*), Maringá, Paraná, Brasil, 2020

## DISCUSSÃO

A averiguação inicial da AFE, que contempla o índice KMO, a testagem de esfericidade de *Barllett* e o determinante da matriz, apresentou índices satisfatórios para o H-PEPSSbr. Na literatura, existem premissas de que o uso dos referidos indicadores versa para o seguimento da análise fatorial, mediante indicadores que correspondam aos valores aceitáveis^(33)^.

Na integração da AFE com a TRI, no modelo comparado para a dimensionalidade na versão reduzida (16 itens) e na versão completa (23 itens), no que se refere à análise paralela (AP), constatou-se uma estrutura unidimensional. No estudo original, os autores sustentaram a escolha da AFC e não da AFE sob o argumento “uma abordagem de análise fatorial confirmatória foi considerada mais apropriada do que a análise fatorial exploratória”^(5)^. Essa afirmação não tem fundamentação técnica na literatura psicométrica de ponta, havendo inclusive recomendações extensivas sobre a importância da exploração do modelo via AFE^(5)^.

Na testagem da versão reduzida e completa para seis dimensões, conforme o modelo teórico, constataram-se problemas como presença de *cross loadigns* e discriminação não aceitável, tanto no ambiente de sala de aula como na prática clínico-assistencial. Segundo a literatura, a presença dos *cross loadigns* é um problema que acumula evidências à inadequação do modelo^(34)^ ao indicar que houve carregamento fatorial aceitável para mais de uma dimensão/fator, representando relação entre fatores diferentes. Neste sentido, os itens devem ser considerados para a análise de retenção ou extração da matriz^(33)^.

A presença da discriminação dos itens também resultou em valores abaixo dos aceitáveis. Estes resultados indicam que o modelo de seis dimensões não mede a força de associação entre o item e a variável latente. Também foi atestada a unidimensionalidade pela AP, corroborada pela associação dos métodos BIC e HULL. Ressalta-se que a associação da testagem de dimensionalidade versão reduzida do H-PEPSS*br* com outros métodos (BIC e HULL) confirmou a unidimensionalidade do modelo.

É versado na literatura que o uso da AP é mais indicado para dados que não seguem a normalidade^(42,43)^, o que é comum em escalas do tipo *Likert*. No entanto, a associação a outras técnicas amplia a robustez das análises de dimensionalidade, como foi apresentado neste estudo. Cita-se que a BIC possibilita a aproximação de modelos, baseada na estatística bayesiana, permitindo comparar o modelo entre modelos hipotéticos^(39)^. Já a HULL busca o equilíbrio entre o ajuste do modelo e os graus de liberdade^(40)^.

Nesta investigação foram empregadas técnicas contemporâneas e completas, com uso de indicadores não usuais em estudos psicométricos. Alguns dos indicadores, como o UNICO, ECV e MIREAL, são recentes e não se encontram disponíveis em *softwares* comerciais. O uso da validade cruzada também possibilita a análise robusta dos resultados iniciais da exploração dos dados com base em um conjunto de dados adicionais independentes para orientar fortemente o processo analítico^(51)^.

No escopo das cargas fatoriais do modelo, a versão reduzida unidimensional do H-PEPSSbr não apresentou problemas/inconformidades de multicolinearidade e *heywood cases.* A multicolinearidade ocorre quando as cargas fatoriais ficam acima de 0,85 e podem indicar redundância dos itens, problemas de distribuição de dados, e gerar distorções na mensuração da variável latente. Já *heywood cases* ^(28)^ ocorrem quando as cargas são violadas da variação de - 1 a +1, relacionado às inadequações da amostra, estimação imprópria da variância de erro e indefinição do modelo. A ausência desses vieses de interpretação permite afirmar que não foram constatados efeitos deletérios de amostra e de modelo.

A discriminação dos itens, baseando-se na aplicação da TRI, apontou índices satisfatórios, atestando a confiabilidade do instrumento, reforçando e legitimando os resultados obtidos nos indicadores das análises fatoriais. Na perspectiva da confiabilidade, os valores de Alfa e Ômega se apresentaram com valores satisfatórios, entre 0,93 e 0,94. Os valores de *Alfa de Cronbach* encontrados neste estudo foram superiores aos relatados no estudo de Ginsburg^(5)^, que variaram entre 0,81 e 0,85 nas seis dimensões relatadas.

Neste estudo, a AFE ou modelo irrestrito visa identificar uma estrutura em um conjunto de itens, sem estabelecer relações *a priori* sobre a estimação do número de componentes a serem extraídos^(32)^. Por sua vez, o emprego da TRI integrada a outras técnicas possibilita a combinação de mais de um método de avaliação, com uso de técnicas robustas^(35)^. Este consegue avaliar a probabilidade de o modelo ser estável entre estudos, populações ou subpopulações^(41)^

O EAP *marginal reliability* também indicou valores que representam a confiabilidade dos *scores* do instrumento. Isso porque, segundo a literatura, índices satisfatórios de EAP apontam a qualidade das estimativas resultantes dos *scores* do instrumento^(41,49)^. Entretanto, ressalta-se que há necessidade de ampliação das testagens sobre a parametrização do teste.

Em um estudo^(11)^ foi apresentado um instrumento final de 38 itens, e em três estudos^(8,12,14)^ houve a apresentação do H-PEPSS por meio de 23 itens, indicando diversas variações de configuração do instrumento, com uso de diversos índices de validade, que apresentam fragilidades. Houve emprego exclusivo de Alfa de Cronbach^(11)^, AFC associada à validação externa, convergente e discriminante^(9)^, AFC com validação discriminante^(10)^, AFC^(5,12,15)^e AFE^(14)^ ou, ainda, associação da AFC e AFE^(8,13)^.

### Limitações do estudo

A versão brasileira do H-PEPSSbr é o primeiro estudo a relatar uma versão unidimensional, apoiada em técnicas contemporâneas que evidenciam a validade de estrutura interna deste modelo. As fragilidades atinentes às competências socioculturais pouco difundidas de forma integralizadora em nossa realidade cultural podem estar relacionadas a não conformidade de um modelo multidimensional da versão brasileira. O desconhecimento de outras escalas para o mesmo fim na realidade brasileira, até o momento, apresenta-se como limitação deste estudo para possíveis comparações. Recomenda-se que, a partir da consolidação das competências socioculturais de segurança do paciente na realidade brasileira, busque-se avançar na exploração da validade de associação com outras variáveis e consequências do teste.

### Contribuições para a área da Enfermagem

A testagem da estrutura interna da versão brasileira do H-PEPSSbr demonstrou que o instrumento consegue avaliar a confiança autorrelatada de estudantes de Enfermagem acerca das competências socioculturais de segurança do paciente, e apresentou evidências de que é um modelo unidimensional com confiabilidade e discriminação satisfatórias. O instrumento constitui uma ferramenta importante no planejamento e/ou monitoramento de ações voltadas à promoção da segurança do paciente em instituições de formação superior e cenários da educação permanente. Além disso, fomenta a necessidade institucional de inclusão do referido tópico no currículo multiprofissional de saúde na realidade cultural nacional, de maneira mais congruente e padronizada.

## CONCLUSÕES

A versão brasileira do H-PEPSSbr mostrou ser unidimensional, obtendo índices de evidência de validade para a estrutura interna entre estudantes de Enfermagem, com evidência de replicabilidade para outros contextos educacionais da área de saúde. A integração de técnicas contemporâneas demonstra avanço científico e originalidade no processo de validação.

## Data Availability

Os dados de pesquisa não estão disponíveis.
